# Melancholia before the twentieth century: fear and sorrow or partial insanity?

**DOI:** 10.3389/fpsyg.2015.00081

**Published:** 2015-02-03

**Authors:** Diogo Telles-Correia, João Gama Marques

**Affiliations:** University Clinic of Psychiatry and Psychology, Faculty of Medicine, University of Lisbon, Lisboa, Portugal

**Keywords:** melancholia, depression, psychopathology, history, eighteenth century, history, nineteenth century

## Abstract

Throughout the history of psychopathology, several meanings have been assigned to the term melancholia. The main ones were related to affective disorders (fear and sadness) and abnormal beliefs. At the time of Hippocrates melancholia was regarded mainly in its affective component. Since that time, and until the eighteenth century, authors and opinions have been divided, with both aspects (affective disorders and abnormal beliefs), being valued. Finally, in the eighteenth to nineteenth centuries, with Pinel at its peak, melancholia becomes exclusively a synonym of abnormal beliefs. At the turn of the nineteenth to the twentieth century, the affective component returns as the main aspect characterizing melancholia.

## INTRODUCTION

Throughout the history of psychopathology, many terms have been introduced. Some have disappeared, as “*Phrenitis*” and “*Carum*,” while others have survived (Berrios, 1996a). Among the surviving terms, some have kept their original meaning, while others have seen their definition change. “Melancholia” is an example of the latter: since its appearance its meaning has differed widely from the current one.

Burton, who dedicated a book to the phenomenon in the seventeenth century, *The Anatomy of Melancholy*, states that: “The tower of Babel never yielded such confusion of tongues as the chaos of melancholy doth variety of symptoms” (Burton, 1883, p. 240).

Throughout history, the two most important meanings of melancholia had to do with affective disorders (mainly depressive mood), and abnormal beliefs. Since Hippocrates, the authors dealing with the subject have alternated between one kind and the other of the symptoms.

Existing work on the subject tends to prefer one side of the two: either insisting that the affective side of melancholia has been neglected before the twentieth century (Berrios, 1996a; Stanley, 1983), or else stating that this aspect has always been paramount (Radden, 2000).

This article aims at reviewing the contributions by the different authors to the construction of the current term melancholia, throughout history, holding the view that both affective and disorders and abnormal beliefs have been valued (and not one of them exclusively).

## FROM HIPPOCRATES TO THE EIGHTEENTH CENTURY

Hippocrates (460–379 BC) attributed the melancholic state (as well as other disorders such as dysentery, skin rashes, etc.) to the excess of black bile, one of the four basic humors described by the author (blood, black bile, yellow bile, and *phlegm*) (Hippocrates, 1923). According to the author, these states were clinically characterized by several symptoms, above all fear and sadness.

Galen (129–216 AD) followed Hippocrates in his theory of temperaments, referring four types of temperament resulting from humoral excesses: melancholic (black bile) (in this case, melancholy was the greek name, *atrabilia* the latin name), optimistic (blood), choleric (yellow bile), and phlegmatic (“*phlegm”*) (Galen, 1952).

For Galen, along with depressive symptoms (“all patients presented fear and despondency”), melancholic patients showed bizarre and fixed ideas (probably alike to what we call today “delusion”) and a behavior which was consistent with such ideation: “There are patients who think to have become a sort of snail so that they must escape everyone in order to avoid having their shell crushed, while others fear that Atlas, who supports the world, may grow weary and vanish, among other imaginary ideas” (Galen, 1976). On the other hand, Galen states that these ideas are restricted to a theme, the remaining life of the patient being unaffected: “although he was able to discuss other issues reasonably and recognise the people who were present, he kept believing that some flutists were constantly playing next to his house…” (Galen, 1833).

Aretaeus of Cappadocia (First Century AD), regarding melancholic patients, states that: “Many fear to be given poison to drink; their senses redouble in penetration and acuteness rendering them suspicious and able to an extreme of discerning hostility everywhere” (Trélat, 1839, p. 73–82).

He characterized this illness through two fundamental dimensions: (1) as an emotional phenomenon, consisting of a state of anguish (*animi angor*); (2) as an intellectual phenomenon consisting of a delusional conception that absorbs and fixes the mind (“*in una cogitatione defixus*”) (Trélat, 1839).

The notion of a partial delusion was to become dominant in the authors dealing with the subject after Aretaeus, and until the end of the eighteenth century.

Andreas Laurentius (1560–1609) was the author of an important revision of the meanings assigned to the concept of melancholia from Galen to the sixteenth century. He defines melancholia as the presence of “delirium with no fever, but with fear and sadness” (Laurentius, 1599). He adds that “all melancholic patients have a disturbed imagination,” and dedicated a great part of his work specifying the type of delusions observed in these patients (fear that Atlas would drop the world, the idea of having been beheaded or swallowed by a serpent, etc.) (Laurentius, 1599). On the other hand, he stresses that “apart from these ideas their imagination remains undisturbed and they are able to speak marvellously of all other issues” (Laurentius, 1599). Laurentius believed that this pathology was caused by an excess of black bile (in the like of Hippocrates and Galen). “The coldness and darkness of this humour affect the mind, especially the imagination” (Laurentius, 1599).

Bright (1551–1615), in his *Treatise of Melancholy*, which is said to have inspired Burton in his later work, states in regard to melancholic patients that they “are for the most part sad and fearful, and such as rise of them: as distrust, doubt, diffidence, or despair” (Bright, 1586).

Burton (1577–1640) tries to gather in his work a summary of all the meanings of melancholia upheld to that date, comparing such a task to “capturing many-headed beast” (Burton, 1883, p. 51). Even so, Burton tries to consistently unify the description of the various authors.

He states that patients suffering from melancholia present multiple symptoms, which “may be infinite,” the most frequent being fear and sorrow (“Fear and sorrow are the true characters and inseparable companions of most melancholy”) (Burton, 1883, p. 109). However, among the several symptoms described are included ideas of persecution, poisoning and jealousy. He refers that these patients can be “most violent in all their imaginations, not affable in speech.” But he stresses that these ideas are not generalized but contained within specific boundaries, as “they are of profound judgement in some things, although in others, non *recte judicant inquieti*” (Burton, 1883, p. 283).

William Cullen (1710–1790) uses the expression “partial insanity” to define the monothematic delusion in melancholia, contrasting with “universal insanity,” which he equates to mania. Nonetheless, the author highlights the difficulty of drawing a line between the two situations: “the boundaries between universal and partial insanity cannot always be drawn with accuracy” (Cullen, 1793, p. 76).

For this reason, Thomas Willis (1621–1675), prefers to divide melancholia in two types: the universal type (in which the “delusion” is extensive to almost anything) and the particular type (in which the judgement of the individual is affected only in one or two areas) (Willis, 1683).

Sennert (1572–1637) defined melancholia as: “a concentration of the soul upon the same idea or a delusion acting on a false thought which is almost exclusive,” “whose judgement is little changed or only in what regards one object.” This author included in melancholic cases states of sadness or joy, as “in melancholy the delusion is sometimes joyful” (Trélat, 1839).

Sauvages (1706–1767) held a similar view, stating that “melancholia is characterised by exclusive delusion,” while Lorry (1726–1783), refers to melancholia as “a partial delusion inflated with exciting passion” (De Matos, 2007, p. 25).

In the beginning of the nineteenth century, Benjamin Rush (1746–1813) described two varieties of partial insanity: tristimania and amenomenia. While the former replaced the term “hypochondria” (when the delusional ideas refer to the patient, their condition or matters and are painfully lived), the latter replaced the term “melancholia” (when the delusions refer to objects outside the patient and are lived with pleasure—or absence of pain). It should be stressed that at the time hypochondria was often regarded as a slight form of melancholia and not as it is seen today (Rush, 1827).

Pinel (1745–1826) abandons the humoral theory in favor of a more rigorous descriptive psychopathology, narrowing down mental disorders to four main groups: Melancholia, Mania, Idiocy, and Dementia (Pinel, 1806). In melancholia, the patients are “overwhelmed by an exclusive idea, endlessly recalled in their words, which seems to absorb all their faculties.” It is distinguished from mania, “nervous excitation or extreme restlessness, sometimes to the point of fury, and a variable level of general delusion” (Pinel, 1806, p. 150).

For Pinel, melancholia takes on two opposite forms: (1) “a heightening of pride and the chimeric idea of possessing infinite richness and power without limits”; (2) “the most fearful despondency, a profound dejection or even despair, therefore considering two forms of melancholia: depressive and expansive” (Pinel, 1806, p. 136).

Hence, until the eighteenth century, most authors privileged abnormal beliefs to affective disorders in melancholia. Aretaeus’ “*angor animi*” had disappeared leaving only the concept of the mind absorbed “*in una cogitatione*.”

## AFTER THE EIGHTEENTH CENTURY

One of the most important steps for the change taking place in the turn of the eighteenth to the nineteenth century was the work of Esquirol (1772–1840), who labeled “monomania” several situations of partial insanity, characterized by delusion which was limited to one object or to a restricted number of objects: “I propose the word monomania a term which express the essential character of that form of insanity, in which the delirium is partial, permanent, gay, or sad” (Esquirol, 1845, p. 200). “Regarding affectivity, they take on two forms: depressive (lypemania), and expansive (monomania itself)” (Esquirol, 1845, p. 202).

According to Esquirol himself, “lypemania” is very similar to Rush’s “tristimania” and to Pinel’s “depressive melancholia”, while “monomania” is similar to Rush’s “amenomania” and to Pinel’s “expansive melancholia” (Esquirol, 1845).

Therefore, partial insanity is no longer comprehended in the term “melancholia” and is given a new designation, that is, “monomania.” Esquirol states that: “writers have confounded monomania with melancholia because in both the delusion is fixed and partial.” Within “monomania,” a specific type may be distinguished due to its depressive affective content, “lypemania,” which Esquirol uses as a synonym for “melancholia,” (“lypemania or melancholia”) throughout his work. In this way, melancholia regains its connotation of a depressive state. The further disappearance of monomania causes the term “lypemania” to fall in disuse, while “melancholia” remains in use.

Two of Esquirol’s disciples, Falret (1794–1870) and Baillarger (1809–1890) coined the terms “circular insanity” (“folie circulaire”) and “dual form insanity” (“folie à double forme”), based on the affective characteristics and motor activity in which manic excitement alternates with mental depression. These would come to inspire Kraepelin’s nosology (Radden, 2000).

Tuke (1827–1895), in his compendium “A dictionary of psychological medicine, giving the definition, etymology and synonyms of the terms used in medical psychology, with the symptoms, treatment, and pathology of insanity,” edited in 1892, points out affective symptoms as paramount in melancholia: “A disorder characterized by a feeling of misery which is in excess of what is justified by the circumstances in which the individual is placed” (Tuke, 1892, p. 787). He also refers the frequent presence of delusion: “as a rule, the disorder of feeling is accompanied, with more or less evidence, by a disorder of thought, and actual delusion accompanies the melancholia” (Tuke, 1892, p. 789). This delusion could take on any type. In Tuke’s dictionary are included other reviewed concepts, such as “monomania” or “circular insanity,” faithful to the designations of the original authors. Curiously, in Tuke’s dictionary, the term “mental depression” is often quoted as an antonym of exaltation and a synonym of simple melancholia, without delusion, “characterised by the chief symptoms of simple melancholia; the patients look sad without having melancholic delusions” (Tuke, 1892, p.240). This term, with a physiological connotation, will be diffused until the twentieth century (Berrios, 1996b).

At the end of the nineteenth century, kraepelinian nosology separates affective disorders (Manic Depressive Illness, including melancholia) from Dementia Praecox. Kraepelin overvalues affective symptoms in the former and thought and cognitive changes in the latter. Therefore, at the turn of the nineteenth century, the idea of melancholia as a disorder mostly affecting the abnormal beliefs restricted to some objects is gradually abandoned in favor of a disease mostly characterized by affective symptoms, namely depressive ones.

In the beginning of the twentieth century the term melancholia was gradually displaced by the term depression, which had a physiologic connotation, showing up in the medical manuals as “mental depression” (Berrios, 1996b).

Nowadays the term melancholia is reserved to certain cases of severe depression, usually known as endogenous depression. This kind of depression is characterized by the presence of profound sadness, anhedonia, loss of emotional resonance, vegetative symptoms (insomnia, anorexia, circadian variability in mood), a seasonal pattern, motor retardation and presence of delusions and/or hallucinations. It is thought that the endogenous depression has mainly an organic cause (with several neurobiological alterations, including psychoimmunological), and a better response to medication (and in severe cases electroconvulsive therapy) than the reactive depression (also called neurotic or situational depression) (Telles-Correia, 2012).

It is in this perspective that the term melancholia appears in the DSM-IV and DSM-5, as “depression with melancholic features.” In the ICD-10 the term melancholia is not present anymore.

## DISCUSSION AND CONCLUSIONS

Since its appearance, the term “melancholia” has suffered an evolution in its meaning.

At the time of Hippocrates, “melancholia,” resulting from an excess of black bile, was a state characterized by affective dimensions: fear and sadness.

Galen upheld Hippocrates’ physiopathological theory of melancholia. However, apart from the affective characteristics, he also recognized fixed and bizarre ideas, restricted to a theme, with the other mental functions being preserved.

Aretaeus of Cappadocia, his contemporary, also upheld the idea that melancholia consisted of an emotional (*animi angor*) and intellectual (*in una cogitatione defixus*) phenomenon.

**FIGURE 1:**
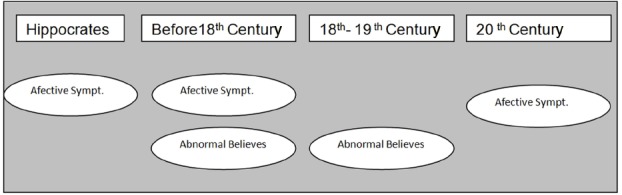
**Evolution of the meaning of the term melancholia throughout history**.

The idea of a partial delusion gradually became dominant in the characterization of melancholia in the authors who came after Galen until the end of the eighteenth century. This conception reaches its peak with Pinel, who distinguishes “mania” (accompanied by general delusion) from melancholia (accompanied by exclusive delusion; Figure 1).

At the turn of the nineteenth to twentieth century, the affective component is recovered as the main change characterizing melancholia. With Esquirol naming the cases of partial insanity “monomania” (the term “melancholia” being abandoned for this meaning), and later, the definition of the cases of manic excitement and cyclic mental depression by Falret, Baillarger, and Kraeplin, the affective component of melancholia would be revalued (Figure 1).

We thus conclude that throughout its evolution, “melancholia” has been associated to two fundamental meanings: affective disorders and abnormal beliefs. Some have minimized the importance of the affective component before the twentieth century. However, we agree with Radden, as from Hippocrates to the present day, many authors valued affective symptoms such as sadness, grief and fear in the definition of “melancholia” (Radden, 2000).

It is our opinion that studies reflecting on the evolution of concepts in psychopathology are important. Studying the history of psychopathology is a powerful way of calibration, by which language in Psychiatry can be improved and prepared for more rigorous quantification.

The epistemology of psychopathology “has to include a combination of methods as history, philosophy and empirical investigation” (Berrios, 2011, p. 39). The history of psychiatry and psychopathology brings to us some information about the social processes where concepts have evolved, philosophy clarifies if the language used is sufficiently powerful, and empirical investigation tests the validity of the new concepts toward reality (Berrios, 2011).

### Conflict of Interest Statement

The authors declare that the research was conducted in the absence of any commercial or financial relationships that could be construed as a potential conflict of interest.
